# Mechanically rigid supramolecular assemblies formed from an Fmoc-guanine conjugated peptide nucleic acid

**DOI:** 10.1038/s41467-019-13250-x

**Published:** 2019-11-20

**Authors:** Vasantha Basavalingappa, Santu Bera, Bin Xue, Ido Azuri, Yiming Tang, Kai Tao, Linda J. W. Shimon, Michael R. Sawaya, Sofiya Kolusheva, David S. Eisenberg, Leeor Kronik, Yi Cao, Guanghong Wei, Ehud Gazit

**Affiliations:** 1 Department of Molecular Microbiology and Biotechnology, George S. Wise Faculty of Life Sciences, Tel Aviv University, 69978 Tel Aviv, Israel; 2 Collaborative Innovation Center of Advanced Microstructures, National Laboratory of Solid State Microstructure, Key Laboratory of Intelligent Optical Sensing and Manipulation, Ministry of Education, Department of Physics, Nanjing University, 210093 Nanjing, People’s Republic of China; 3 Department of Materials and Interfaces, Weizmann Institute of Science, 76100 Rehovoth, Israel; 4 Department of Physics, State Key Laboratory of Surface Physics, Key Laboratory for Computational Physical Sciences (MOE), Fudan University, 200433 Shanghai, People’s Republic of China; 5 Department of Chemical Research Support, Weizmann Institute of Science, 76100 Rehovoth, Israel; 6 Howard Hughes Medical Institute, UCLA-DOE Institute, Departments of Biological Chemistry and Chemistry and Biochemistry, University of California, Los Angeles, Los Angeles, CA 90095 USA; 7 Ilse Katz Institute for Nanotechnology, Ben Gurion University of the Negev, 84105 Beer Sheva, Israel

**Keywords:** Chemistry, Materials science, Nanoscience and technology

## Abstract

The variety and complexity of DNA-based structures make them attractive candidates for nanotechnology, yet insufficient stability and mechanical rigidity, compared to polyamide-based molecules, limit their application. Here, we combine the advantages of polyamide materials and the structural patterns inspired by nucleic-acids to generate a mechanically rigid fluorenylmethyloxycarbonyl (Fmoc)-guanine peptide nucleic acid (PNA) conjugate with diverse morphology and photoluminescent properties. The assembly possesses a unique atomic structure, with each guanine head of one molecule hydrogen bonded to the Fmoc carbonyl tail of another molecule, generating a non-planar cyclic quartet arrangement. This structure exhibits an average stiffness of 69.6 ± 6.8 N m^−1^ and Young’s modulus of 17.8 ± 2.5 GPa, higher than any previously reported nucleic acid derived structure. This data suggests that the unique cation-free “basket” formed by the Fmoc-G-PNA conjugate can serve as an attractive component for the design of new materials based on PNA self-assembly for nanotechnology applications.

## Abstract (web summary)

DNA is an attractive nanomaterial, yet limited compared to polyamides due to low stability and mechanical issues. Here, the authors combine polyamide and DNA characteristics to form mechanically rigid self-assembled peptide nucleic acid based materials with high stiffness and Young’s modulus.

## Introduction

Among the basic set of molecular building blocks naturally comprising biological systems, deoxyribonucleic acid (DNA) is fundamental and plays an essential role in the storage and replication of genetic information^1,2^. DNA self-associates into various inter-molecular and intra-molecular secondary structures including the canonical double helical structure and the non-canonical G-quadruplex and i-motif structures, comprised of particular nucleic acid sequence motifs^3,4^. The variety and complexity, along with chemical robustness^5^, make DNA-based building blocks interesting components for nanotechnology^6–8^. However, the application of naturally derived DNA-based structures in material science is limited by their low stability and mechanical rigidity, with Young’s moduli of 0.3–2 GPa^9–18^. Such Young’s modulus values are significantly lower compared to amino acids and polyamide-based materials, such as peptides, proteins, poly-paraphenylene terephthalamide (Kevlar®), poly(hexamethylene adipamide; Nylon 66®), and amyloid-based materials, where Young’s modulus can be as much as two orders of magnitude higher^19–28^. This suggests that assembly of a polyamide backbone in the structural pattern of DNA could pave the way for tailored design of novel synthetic materials offering high mechanical rigidity.

A promising building block for this goal is PNA, a synthetic mimic of DNA with a neutral polyamide backbone^29,30^. PNAs show structural features similar to those of DNA and have been suggested for potential applications in fields ranging from biology and chemistry to materials science^31–33^. Specifically, PNAs are capable of hybridizing to DNA and RNA molecules with high affinity and specificity, resulting in duplex and triplex structures^34,35^. PNAs alone have the potency to fold into quadruplexes when enriched with guanine residues^36,37^. Also, among fully synthetic G-quartets, PNA-derived assemblies are known to catalyze peroxidase-mimicking reactions similar to natural G-quadruplex-based DNAzymes^38,39^. Recently, we and others have demonstrated the self-assembly of Fmoc-protected guanine-containing PNA-dipeptides, PNA-monomers and PNA conjugates into various supramolecular structures, ranging from Watson-Crick base-pairing to nanospheres and photonic crystals with interesting optical properties^40–43^. The Fmoc group is known to induce excellent self-assembly by increasing π-π stacking and hydrophobic interactions^42,44^. Also, fluorene-derivatives have been used as blue or green emitters in optoelectronic devices^45^. However, characterization of the mechanical properties of these PNA-based nanostructures is lacking.

Here, we aimed to obtain mechanically rigid self-assembled nanostructures, inspired by nucleic acids. For this purpose, we designed an Fmoc-G-PNA conjugated to a hydrophobic moiety, which increases the conformational space for folding by extending the chain length. This Fmoc-G-PNA conjugate self-assembles into various supramolecular structures with diverse morphologies. Interestingly, it forms head-to-tail hydrogen bonds between guanine of one molecule and the Fmoc group of another, thereby forming a cyclic tetrameric structure (Fmoc-G-PNA tetramer). Owing to its design, the Fmoc-G-PNA tetramer demonstrates a high Young’s modulus of 17.8 ± 2.5 GPa, along with fluorescent properties. The high mechanical rigidity and interesting photoluminescent features suggest that the self-assembled structures formed by the Fmoc-G-PNA tetramer may serve as components in the design of PNA-derived materials and as a starting point for future nanotechnology and optoelectronics applications.

## Results

### Synthesis and self-assembly of the Fmoc-G-PNA conjugate

**Fig. 1:**
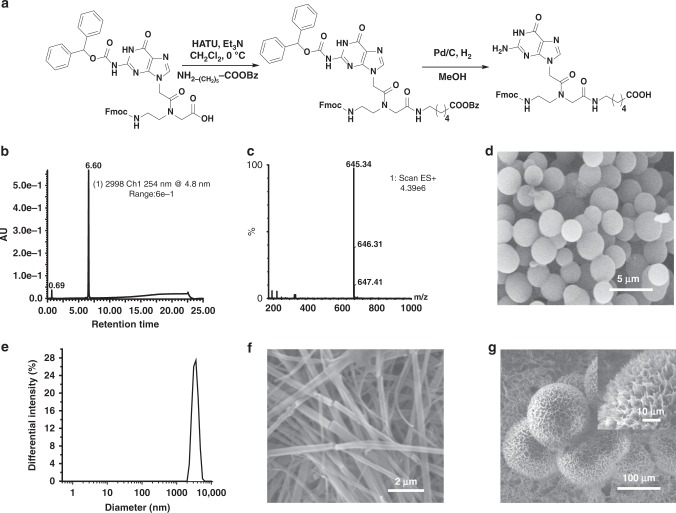
Synthesis of the Fmoc-G-PNA conjugate and self-assembly characterization. **a** Schematic representation of the synthetic procedure. **b** LC profile and **c** the corresponding mass spectrum. **d** SEM image of the microspheres. **e** DLS analysis of the spheres. The highest differential intensity was observed for a diameter of 3.39 μm. **f** SEM image of the elongated nanoribbons. **g** SEM image of the microparticles formed in a hot aqueous solution

The hydrophobic Fmoc-PNA conjugates of adenine, guanine, and cytosine were synthesized using a solution phase peptide synthesis protocol (a representative synthetic scheme for the Fmoc-G-PNA conjugate is shown in Fig. 1a). The final products were purified through high-performance liquid chromatography (HPLC) and characterized by mass spectrometry (LC-MS, Fig. 1b, c and Supplementary Figs. 1–4) and nuclear magnetic resonance (NMR) spectroscopy (Supplementary Figs. 5–7). To identify the suitable conditions for the self-assembly of the PNA conjugates into ordered structures, we used the solvent switch method. Generally, PNAs are insoluble in water at ambient temperature, but highly soluble in methanol. This property was utilized for triggering the self-assembly, whereby a concentrated MeOH stock solution of each PNA conjugate was diluted into water at varying water/MeOH ratios or concentrations. Under these conditions, only the G-PNA conjugate formed well-ordered supramolecular structures (Fig. 1), while adenine and cytosine PNA conjugates did not self-assemble into ordered nanostructures (Supplementary Fig. 8), in line with previous reports^40,41^. Up to a concentration of 0.1 mg/mL in 7/3 methanol/water (v/v), the G-PNA conjugate self-assembled into spheres (Fig. 1d). Scanning electron microscope (SEM) image (Fig. 1d), as well as dynamic light scattering (DLS) measurement (Fig. 1e), revealed the average diameter of the spherical assemblies to be 2–4 µm. Raising the concentration above 0.5 mg/mL in 7/3 methanol/water (v/v) surprisingly altered the morphology to nanoribbon-like assembles, as observed by SEM (Fig. 1f). In pure aqueous solution, the G-PNA was found to self-assemble when dissolved at a concentration of up to 0.1 mg/mL by heating the solution at 100 °C for 5 min, followed by vigorous shaking. When the partially dissolved hot solution was filtered and cooled to room temperature, an immediate increase in turbidity was observed in the clear filtrate solution. SEM micrograph analysis showed the presence of spherical microparticles 100–150 μm in diameter (Fig. 1g). Notably, the morphology of the microspheres comprised a very rough surface and distributed micropores (Fig. 1g, inset).

### Single crystal analysis of the Fmoc-G-PNA conjugate

**Fig. 2:**
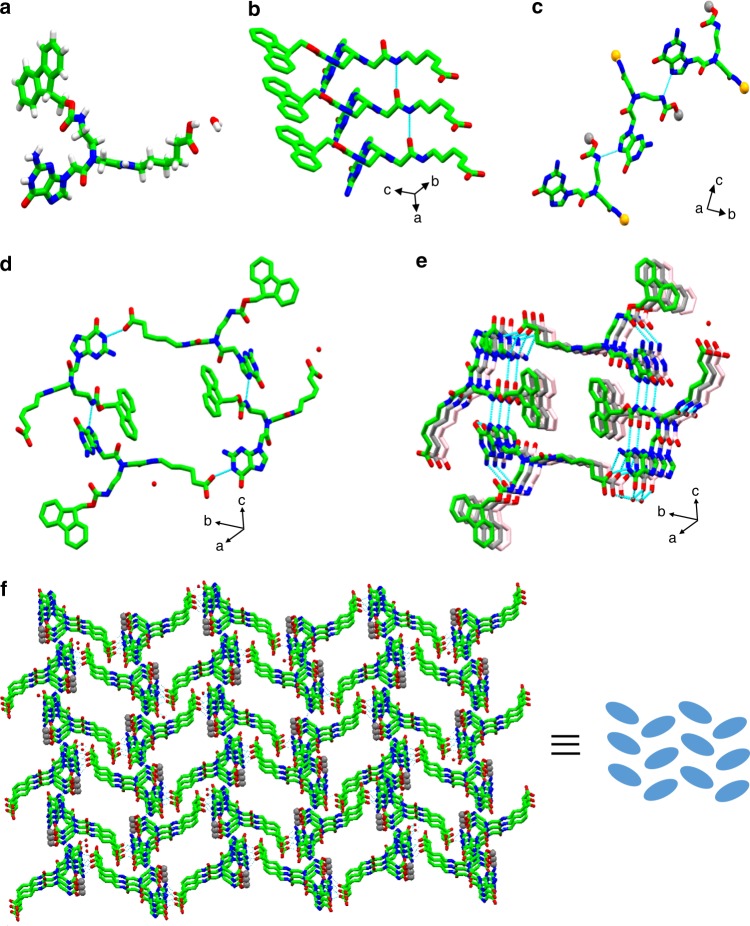
Single crystal structural analysis of the Fmoc-G-PNA tetramer. **a** The asymmetric unit. **b** Supramolecular parallel array of the asymmetric unit, obtained through intermolecular H-bonds and aromatic-aromatic interactions. **c** Truncated view of a ribbon-like structure formed in the crystallographic $\vec c$-direction. The Fmoc group and aliphatic chains are represented by gray and orange spheres, respectively. **d** Non planar cyclic tetrameric structure. **e** Quadruplex inspired superstructure view down crystallographic $\vec a$-direction. **f** Herringbone-like arrangement of the G-wire in a higher-order structure produced by lateral orientation. The H-bonds are shown in light blue dashed lines and the Fmoc rings are represented by a gray sphere

To understand the molecular packing and the interactions of the assemblies formed by Fmoc-G-PNA conjugate, we produced single crystals, allowing for structure determination at a 0.8 Å resolution (Supplementary Fig. 9). The X-ray crystallographic analysis revealed that G-PNA conjugate crystalized in P2_1_/c space group, with one G-PNA monomer and one water molecule in the asymmetric unit (Fig. 2a, Supplementary Fig. 10). The Fmoc group adopted a *syn* conformation and the aliphatic tail resided in an *anti-*orientation with respect to the heterocyclic base guanine^4^. No intramolecular hydrogen bond was found in the structure. In the crystallographic $\vec a$-direction, individual subunits of the PNA molecules were stacked in a parallel orientation, stabilized through an intermolecular H-bond (N_BJ_-H_BJ_^**…**.^O_BI_), as well as aromatic interactions mediated by the Fmoc group (Fig. 2b). The *anti*-conformation of the aliphatic tail facilitated the growth of a ribbon-like structural arrangement in the $\vec c$-direction through H-bonding between an amide in the aliphatic tail and guanine (Fig. 2c), resulting in the ribbon-like structure observed in SEM imaging (Fig. 1f). This structural arrangement is consistent with the self-assembly of previously reported guanosine/deoxy-guanosine derivatives in the absence of any templating cation^46,47^.

The Fmoc group plays an important role in the self-assembly of the G-PNA conjugate into a cyclic tetrameric arrangement and it comprises an integral part of the structure by forming head-to-tail H-bonds. The hydrophobic moiety, along with providing the conformational space for folding, contributes to the stabilization of the structure^48,49^. Interestingly, four molecules of the PNA conjugate form H-bonds between aliphatic tails and the Fmoc-2-aminoethyl group of neighboring molecules, to produce a cyclic tetrameric structure, the Fmoc-G-PNA tetramer. Due to the presence of the flexible aliphatic tail, the planarity of the overall cyclic arrangement is disturbed to some extent. This non-planar tetrameric arrangement has the look of four-stranded topology obtained by natural/synthetic G-quartets (Fig. 2d)^47,50,51^. Among the four inter-molecularly hydrogen bonded Fmoc-G-PNA conjugates responsible for tetramer formation, the fluorenylmethyl part of two molecules occupies the space either above or below the tetramer plane, with the hydrophobic tail pointing outwards from the cavity. For the other two molecules, the fluorenylmethyl subunit is positioned outside, approximately in the plane of the cavity (Fig. 2d). The individual Fmoc-G-PNA tetramer structural modules stack vertically to produce a quadruplex-like arrangement (Fig. 2e), in the absence of any stabilizing cations. Furthermore, the stacking distance between two adjacent cyclic tetramers is 3.5 Å, resembling the layer separation of the canonical G-quadruplex structure (3.3 Å)^4^. In the higher order assembly, the G-PNA tetramer were stacked in the crystallographic $\vec a$-direction, resulting in the generation of G-PNA wire nanostructures (similar to guanine nanowires, G-wires^52^). Finally, lateral association of the G-PNA wires results in a herringbone-like structural arrangement (Fig. 2f).

In the conventional G-quartet, Hoogsteen H-bonds are formed between four guanine bases, resulting in a planar cyclic tetrameric arrangement. The G-quartets further stack to produce a G-quadruplex structure, generally stabilized by metal cations, e.g., K^+^, Na^+^, Ba^2+^, or Sr^2+^, inside the core, though exceptions have been reported^53,54^. However, exceptions to this traditional structural pattern have been observed in many natural/synthetic G-quadruplexes^39,55–58^. For example, Krauss et al. reported the crystal structure of a thrombin-bound DNA aptamer, in which the orientation of guanine bases broke the Hoogsteen hydrogen-bonded cyclic pattern, but a G-quartet structure was still produced^55^. Several studies on template-assembled synthetic quartets (TASQ) such as DOTASQ^59^, ^PNA^DOTASQ^60^, PorphySQ^61^, and PyroTASQ^62^, have been reported to manifest unusual cation-independent G-quartet stability due to pre-organization afforded by the cavitand scaffold, exemplifying one of the hallmarks of supramolecular chemistry^48^. In another report, pre-organization of guanine units onto a peptidic scaffold allowed a remarkable stabilization of the G-quartet motif in water, in the absence of any cations^63^. The synthetic G-quartet formed by macrocyclic hexaoxazoles, bearing aminoethyl, aminoethoxyethyl, and piperazinyethoxyethyl side chains, is known to stabilize a telomeric G-quadruplex^49^. Thus, in spite of displaying certain structural differences, synthetic G-quartets have been used as stable substitutes for canonical G-quadruplex. Hence, the observed Fmoc-G-PNA tetramer may be considered as a member in the extended family of synthetic G-quadruplexes.

### **F**luorescent properties of the Fmoc-G-PNA tetramer

**Fig. 3:**
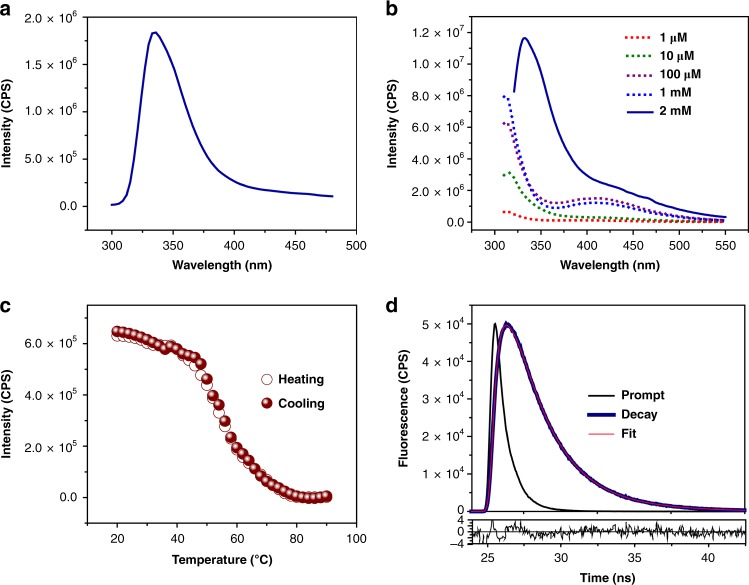
Fluorescence characterization of Fmoc-G-PNA tetramer. **a** Fluorescence emission spectrum of assemblies at λ_ex_ = 280 nm. **b** Fluorescence emission spectra in solution at various concentrations, λ_ex_ = 290 nm. **c** Fluorescence melting profile obtained by monitoring emission at 330 nm under λ_ex_ = 290 nm. **d** Fluorescence decay curve of crystals excited at λ_ex_ = 280 nm and probed at 330 nm, along with the corresponding residuals

Fluorescent characterization of the G-PNA tetramer demonstrated an emission at 330 nm when exited at 260 nm (Fig. 3a). The role of Fmoc group in the fluorescence emission was examined by carrying fluorescence measurements over a range of concentrations. At lower concentrations, an emission peak was observed at 315 nm (λ_ex_ = 280 nm). At higher concentrations, an additional peak at 410 nm appeared, probably due to a different stacking arrangement of the Fmoc groups. However, at 2 mM, the solution turned turbid, the emission peak at 315 nm shifted to 330 nm, and the peak at 410 nm disappeared (Fig. 3b). Overall, the observed fluorescence pattern at higher concentrations was different than those previously reported for parallel or antiparallel orientations of Fmoc self-assembly^42,64,65^. The PXRD pattern of the turbid solution at 2 mM was found to be in good agreement with the PXRD profile of a single crystal of Fmoc-G-PNA tetramer, while at lower concentrations, an amorphous profile was obtained (Supplementary Fig. 11). In contrast the 330 nm peak was absent in the adenine and cytosine PNA conjugates under similar conditions (Supplementary Fig. 12), implies that tetramer structure is formed only in case of G-PNA conjugate. Melting profile of the tetramer, showed a sigmoidal behavior in both heating and cooling cycles (Fig. 3c, Supplementary Fig. 13). Further, to demonstrate the effect of cations, fluorescence measurements were carried out in the presence of Na^+^ and K^+^ ions. No change in the fluorescence emission was observed, as shown in Supplementary Fig. 14. Next, the excited-state dynamics was studied by probing the fluorescence at 330 nm using two different detection techniques—fluorescence spectroscopy and fluorescence lifetime imaging microscopy (FLIM). The Fmoc-G-PNA tetramer possesses tri-exponential decays, from the picosecond to the nanosecond range. Using both techniques, the decay plot was fitted to a tri-exponential decay curve (Fig. 3d, Supplementary Fig. S14a). The tri-exponential fit parameters obtained from fluorescence spectroscopy included three lifetime values (Table 1). The 0.24 ns component with a small fractional amplitude of ∼7% showed ultrafast decay, while the other two components (2.05 ns and 3.53 ns) had fractional amplitude of 49 and 43%, respectively. These differences in fluorescence decay may be attributed to the anisotropic nature and varying size of the crystals, in line with previous reports^66,67^. The fluorescence statistical distribution and lifetime fluorescence image also showed different lifetime values in different parts of the crystals (Supplementary Fig. S15). Quantum yield measurement carried out on the G-PNA tetramer showed a high value of 0.4. Overall, it was observed that the G-PNA tetramer demonstrated fluorescence emission and melting profile similar to that of the canonical G-quadruplex^67–69^. The longer lifetime and high quantum yield values exhibited by the G-PNA tetramer are characteristics of self-assembled Fmoc-PNA peptides^42^. This may be attributed to the combined effect of tetramer formation, Fmoc self-assembly, and hydrophobic interaction.

**Table 1:** Fitting parameters derived from the fluorescence decay of Fmoc-G-PNA tetramer using tri-exponential functions

λ(nm)	a1 (%)	τ1 (ns) ± 0.03	a2 (%)	τ2 (ns) ± 0.03	a3 (%)	τ3 (ns) ± 0.01	τ_avg_ (ns) ± 0.02
330	7.37	0.24	49.37	2.05	43.26	3.53	2.55

The average lifetime τ_avg_ was obtained by (a1τ1 + a2τ2 + a3τ3)

### Analysis of ThT and TmPyP4 binding to Fmoc-G-PNA tetramer

**Fig. 4:**
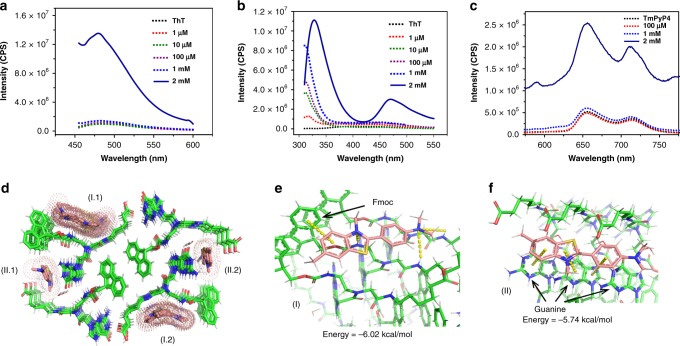
ThT/TmPyP4 binding to Fmoc-G-PNA tetramer. **a** Fluorescence emission spectra of 20 μM ThT with various concentrations of the Fmoc-G-PNA conjugate, λ_ex_ = 440 nm. **b** Fluorescence emission spectra of the Fmoc-G-PNA tetramer in the presence of 20 µM ThT, λ_ex_ = 290 nm. **c** Fluorescence emission spectra of 10 μM TmPyP4 with various concentrations of the Fmoc-G-PNA conjugate, λ_ex_ = 433 nm. **d**–**f** Molecular docking of ThT to the Fmoc-G-PNA tetramer. **d** Binding sites of the first 30 energy-optimized docking patterns. The conformations are clustered into two groups: I.1 and I.2 are equivalent due to the crystal symmetry, as are II.1 and II.2. **e**, **f** Interactions mediating the molecule binding in **e** the first and **f** the second conformation clusters

Thioflavin T (ThT) and 5,10,15,20-tetra(*N*-methyl-4-pyridyl)porphyrin (TmPyP4) are fluorogenic dyes, known to specifically bind to G-quadruplex through fluorescent light up^70–73^. We therefore examined the binding of both ThT and TmPyP4 to the observed G-PNA tetramer. As shown in Fig. 4a, ThT alone is weakly emissive, as also found in the presence of other G-self-assembled structures at lower concentrations. Strikingly, a significant rise in fluorescent intensity was observed in the presence of Fmoc-G-PNA tetramer (Fig. 4a). Similar results were obtained when TmPyP4 binding to the Fmoc-G-PNA tetramer was examined (Fig. 4c). As visualized by confocal fluorescence microscopy, G-PNA crystals stained with ThT showed fluorescence emission when excited at 458 nm. In contrast, non-stained crystals were not fluorescent (Supplementary Fig. S16). Interestingly, when excited at 290 nm, a new fluorescence peak was observed at 469 nm alongside the tetramer signature peak at 330 nm, while no change was detected for other samples (Fig. 3f). This new peak can be attributed to topological transformations in the tetramer network due to ThT binding, owing to structural differences between G-PNA teramer and canonical G-quadruplex.

To obtain further insight into the mechanism of ThT/TmPyP4 binding, molecular docking studies were carried out, as previously reported for canonical G-quadruplex^70,71,74^. For this purpose, Fmoc-G-PNA tetramer (consisting of three-layered tetramers) was used as a receptor and a ThT molecule as a ligand. Thirty separate automated dockings were performed using a Lamarckian genetic algorithm and an empirical binding free energy function. The binding free energy of the resulting conformations varied from −6.02 to −5.68 kcal/mol for ThT and −7.34 to −6.90 kcal/mol for TmPyP4. These conformations could be clustered into two groups, as shown in Fig. 4d and Supplementary Fig. 17. Two favorable binding sites were identified: one located in the space between the Fmoc group and the C-terminal carboxyl group (I.1 and I.2), and the other positioned near the guanine (II.1 and II.2). The lowest energy conformers for each cluster were used for further interpretation of the docking results. As shown in Fig. 4e, the benzothiazole ring on the ThT molecule formed a T-shaped π-stacking with the PNA Fmoc group, while the two ThT nitrogen atoms formed hydrogen bonds with the NH group of the PNA peptide. On the other hand, supplementary Fig. 17b shows that the pyridine ring on the TMPyP4 molecule formed a T-shaped π-stacking with the PNA Fmoc group (1), the positively-charged nitrogen atoms formed hydrogen bonds with the –OH or –NH group of the PNA peptide (2,3), while the –NH group formed hydrogen bonds with the carbonyl group (4). These interactions led to strong binding of ThT and TmPyP4 to the assemblies, with a binding energy of −6.02 kcal/mol and −7.34 kcal/mol, respectively. In the second cluster, the ThT (Fig. 4f) and TmPyP4 (Supplementary Fig. 17c) molecules were positioned parallel to the fiber axis. While each of the three aromatic rings of the ThT molecule formed π-stacking interactions with the guanine group in the PNA peptide, in the case of TmPyP4, two of the positively-charged nitrogen atoms formed hydrogen bonds with the –NH group of the PNA peptide. These bindings were also similarly strong, with a binding energy of −5.74 kcal/mol and −6.79 kcal/mol for ThT and TmPyP4, respectively. These results suggest that the G-PNA tetramer also binds to ThT and TmPyP4, similar to G-quadruplex structures, but with a different binding pattern^70,71^, possibly accounting for the formation of a new peak in the tetramer arrangement and the increase in ThT-related intensity.

### Mechanical stability of the Fmoc-G-PNA tetramer

**Fig. 5:**
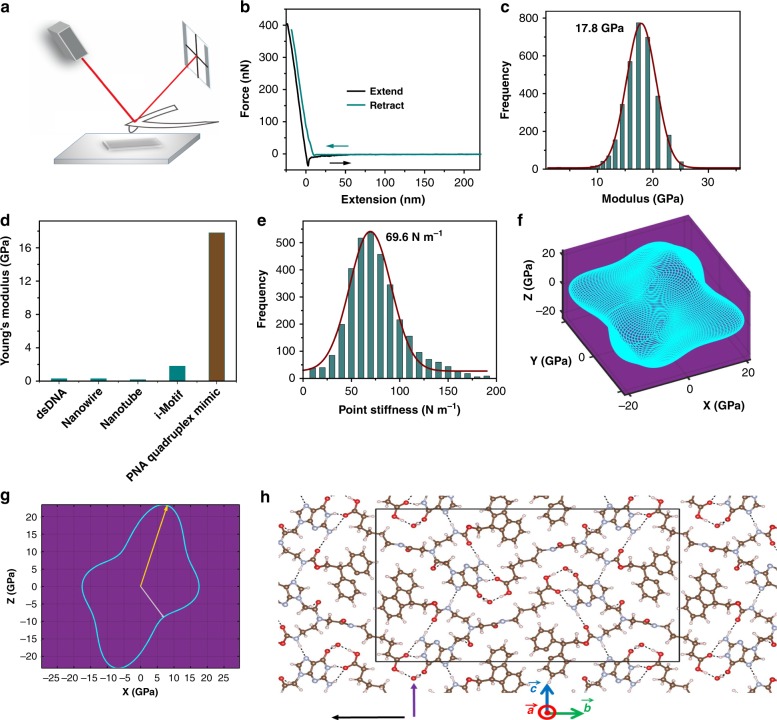
Mechanical properties of the Fmoc-G-PNA tetramer. **a** Schematic illustration of the atomic force microscopy experiments. **b** Typical force–distance traces. The black line corresponds to fitting of the contact region in the “extend” trace using the Hertz model. **c** Statistical distribution of the obtained Young’s modulus. The red line corresponds to the fitting using a Gaussian model. **d** Comparison of Young’s moduli previously reported for various DNA nanostructures and the observed tetramer assemblies in this work. dsDNA from refs. ^9–11^; Nanowire from refs. ^12^; Nanotube from refs. ^13,16,17^; i-Motif from ref. ^14^. **e** Statistical distribution of point stiffness measurements. The red line corresponds to the fitting using a Gaussian model. **f** 3D rendering of computed Young’s moduli. The 3D surface is constructed by setting the distance from the origin to each point on the surface to the value of Young’s modulus in the direction of the vector pointing from the origin to that point. The axes are given in GPa and negative signs are for directional indication only. In this plot, the lattice vector $\vec a$ is on *x*, $\vec b$ is on *y*, and $\vec c$ is in the *x−z* plane. **g** 2D rendering of computed Young’s moduli in the *x−z* plane, using the same lattice vectors alignments as in **f**. Yellow and gray arrows indicate maximum and minimum values, respectively, in this plane. **h** Structure of the crystal. Purple and black arrows indicate the projection directions for minimal and maximal Young’s moduli, respectively, and the projections are in the *y–z* plane. The black frame denotes the projection of a single unit cell in the *y–z* plane

To check the mechanical rigidity of the tetramer assemblies, point-stiffness and Young’s modulus were measured by nanoindentation (Fig. 5a, Supplementary Fig. 18)^75,76^. First, crystals were carefully fixed to a flat glass coverslip. A constant speed of 2 μm s^−1^ was set for the cantilever to approach and retract from the surface of the crystals. Typical force–distance traces are shown in Fig. 5b. The Young’s modulus of the crystal at a given position can be calculated by fitting the approaching traces using the Hertz model. Moreover, the point stiffness could then be calculated according to the force–distance traces. As shown in Fig. 5c, the Young modulus of the crystals was found to be 17.8 ± 2.5 GPa in the *x–z* plane, significantly higher than the values previously reported for DNA self-assembled nanostructures (Fig. 5d)^9–18^. The statistical point stiffness of the tetramer was 69.6 ± 6.8 N m^−1^ (Fig. 5e), higher than most polymers and copolymers^77,78^.

In order to understand the molecular origin of the surprisingly high rigidity of the tetramer assemblies, computational first principles studies were performed using dispersion-augmented density functional theory (DFT)^79–81^. A prerequisite for accurate calculations of mechanical properties is attaining an accurate equilibrium structure^21,27^. As shown in Supplementary Table 1, the computed lattice parameters of the G-PNA crystals were in very good agreement with the experimental crystallographic data, with an absolute average error of 1%. This level of agreement is typical of successful dispersion-augmented DFT calculations^81^. The elastic constant tensor^82–85^ was calculated from stress-strain curves, as previously reported^20,82,83^. Specifically, six different distortions were applied to the unit cell (see Supplementary Details 1). Directional Young’s moduli were then computed from the elastic constant tensor.

As shown in Fig. 5f, g, Young’s moduli along crystallographic axes $\vec a$, $\vec b$, $\vec c$ were computed to be 17.5, 18.6, and 19.0 GPa, respectively, indicating little anisotropy along these directions and showing close approximation to the Voigt and Reuss average Young’s moduli of 22.4 and 19.7 GPa, respectively. While Young’s modulus is quite isotropic along these crystallographic axes, the calculations show that it is not completely isotropic, ranging between a minimum value of 11.2 GPa in the *x*–*z* plane and a maximum value of 28.4 GPa in the *x*–*y* plane (along the directions of the purple and black arrows, respectively, in Fig. 5h). The maximal value of Young’s modulus was found along the direction of hydrogen bonding of the water molecule. This is consistent with a prior study on the directional relation between the hydrogen-bonding network and the Young’s moduli in glycine-based molecular crystals^27^. It is also consistent with previous results on diphenylalanine molecular crystals, showing that water molecules can increase Young’s modulus by as much as 10 GPa^86^. The minimum Young modulus value was found along the direction of shear between phenyl rings and was nearly perpendicular to the hydrogen bonds formed by the water molecules.

Further insights were obtained by comparing the experimental and computational Young’s moduli in the *x*–*z* plane (Table 2), showing very good agreement between theory and experiment and directly confirming the anisotropic nature of Young’s modulus in the plane. Specifically, experimental values in the *x*–*z* plane range between a minimum of 11.0 GPa and a maximum of 26.0 GPa, with an average value of 17.8 ± 2.5 GPa. The 2D rendering of the computed results (Fig. 5g) reveals a minimum of 11.2 GPa and a maximum of 24.6 GPa. The average computed value in the plane is 16.8 GPa, in excellent agreement with the average experimental value.

**Table 2:** Experimental and computed Young’s moduli in the *x−z* plane

	Min	Max	Average
Experiment	11.0	26.0	17.8 ± 2.5
Theory	11.2	24.6	16.8

## Discussion

By combining a polyamide backbone, an aromatic interacting Fmoc group, a nucleic acid, and a hydrophobic chain we have designed an Fmoc-G-PNA conjugate which exhibits supramolecular self-assembly resulting in a rich diversity of structural patterns, from spheres to fibers, depending on the solvent and concentration. In the crystal packing, the guanine base of one molecule alternately H-bonds with the aliphatic tails and Fmoc groups of the next molecules, thereby forming a cyclic tetrameric molecular array, namely a Fmoc-G-PNA tetramer. This structure undergoes further vertical stacking through H-bonds and aromatic interactions, without any stabilizing cations, to produce a unique quadruplex-inspired structure. Surprisingly, the Fmoc-G-PNA tetramer exhibited fluorescence emission, a fluorescence melting profile, and ThT or TmPyP4 binding behavior similar to those of the native G-quadruplex. The fluorescent assemblies of G-PNA tetramer exhibited longer life time and high quantum yield values, similar to peptide or PNA based self-assembled structures. Importantly, nano-indentation experiments performed on these G-PNA tetramer revealed a Young’s modulus of approximately 17.8 ± 2.5 GPa. This high value, confirmed and rationalized by theoretical studies and modeling, makes this material stiffer than any previously reported DNA-based structure.

In conclusion, this work describes a quadruplex-inspired mode of self-assembly for Fmoc-G-PNA conjugates. Combined experimental and theoretical mechanical studies reveal that the tetrameric assemblies possess high mechanical stiffness, as well as significant mechanical stability, compared to natural nucleic acid derived structures. This is due to the special head-to tail packing, additional π–π interactions mediated by the Fmoc groups and aliphatic chains. We envisage that the rigid and fluorescent assemblies formed by the Fmoc-G-PNA conjugate may be utilized as components in the design of novel nanoscale applications and provide a basis for the development of new peptide nucleic acid based materials.

## Methods

### Synthesis of Fmoc-G-PNA conjugate

PNA-conjugates synthesis was carried out using conventional solution‐phase procedures. HATU (Sigma-Aldrich, Rehovot, Israel) (1.0 mmol) was added to a solution of Fmoc-G(Bhoc)-aeg-OH/ Fmoc-A(Bhoc)-aeg-OH/ Fmoc-C(Bhoc)-aeg-OH (PolyOrg Inc. Massachusetts, USA) (1.0 mmol) in CH_2_Cl_2_ (20 mL) at 0 °C. Et_3_N and benzyl ester of 6-Aminohexanoic acid (1.0 mmol) were then added and the solution was stirred until the reaction was complete. Next, the organic layer was washed with 5% Na_2_CO_3_ (2 × 10 mL) and 10% citric acid (2 × 10 mL), water (2 × 10 mL), and brine (2 × 10 mL), and dried over anhydrous Na_2_SO_4_. The solvent was removed under vacuum and subjected to removal of the benzhydroxyl (Bhoc) group. For this purpose, the crude reaction mixture was dissolved using a mixture of EtOAC and methanol (2/1, v/v). Ten percent Pd/C (Sigma-Aldrich, Rehovot, Israel) was then added and the solution was stirred under hydrogen atmosphere at room temperature. After completion, the reaction mixture was filtered using a celite bed and the solvent was evaporated in vacuo to yield the desired product. The final compounds Fmoc-G-aeg-(CH_2_)_5_-OH/Fmoc-A-aeg-(CH_2_)_5_-OH/Fmoc-C-aeg-(CH_2_)_5_-OH, were purified using Waters Pre150 LC reversed phase high‐performance liquid chromatography (HPLC) on an XBridge Prep C18 OBD 5 μm, 19 mm × 100 mm column using an acetonitrile/water system and monitored at 254 nm. LC-MS characterization was performed on Low resolution ESI mass spectrometry Acquity QDa detector coupled with Waters HPLC. ^1^H NMR was recorded by dissolving 5 mg of the compound in 500 µL of DMSO-*d*_*6*_ at 25 °C on a Bruker 500 MHz spectrometer. NMR data were processed and analyzed using Topspin Version 3.5.

### Scanning electron microscopy (SEM)

The G-PNA conjugates were dissolved in a MeOH/water, 7/3 (v/v) mixture at a concentration of 0.1 mg/mL (microspheres) and 0.5 mg/mL (fibers). A 5 μL aliquot of the solution was allowed to dry on a microscope glass cover slip at ambient conditions and was coated with Au. Scanning electron microscopy images were recorded using a JCM-6000PLUS NeoScope Benchtop SEM (JEOL, Tokyo, Japan).

### High resolution scanning electron microscopy (HRSEM)

The Fmoc-G-PNA conjugate was dissolved in double distilled water to a concentration of 0.1 mg/mL by heating the solution to 100 °C under continuous stirring. The partially dissolved hot solution was filtered using a 0.22 μm PVDF syringe filter (Millipore) into a clean beaker with a glass cover slip inside. The turbidity of the solution increased within several minutes as the solution cooled to room temperature, indicating self-assembly. Excess solution was drawn out gently using a syringe. The glass was dried in open air and images were taken using a JEOL JSM 6700F FE-SEM operating at 10 kV, after coating with Cr.

### Dynamic light scattering (DLS)

Eight hundred and fifty microliter sample of the Fmoc-G-PNA conjugate solution was pipetted out into a DTS1070 folded capillary cell (Malvern, Worcestershire, U.K.). The size of the spheres was measured using a Zetasizer Nano ZS analyzer (Malvern Instruments, Malvern, UK) at 25 °C and a backscatter detector (173°). Three measurements were performed and averaged for accuracy.

### Single crystal X-ray diffraction (XRD)

Single crystals of the Fmoc-G-PNA conjugate, suitable for X-ray diffraction, were grown by slow evaporation of the peptide at 2 mM in methanol/water, 7/3 v/v in room temperature. Crystals of diffraction quality were obtained after 2–3 days of sample preparation. G-PNA crystals grew as small needles, ~150 μm long, 10 μm wide and 2 μm thick. X-ray diffraction data set from one crystal was collected at the Advanced Photon Source (Argonne National Laboratory), beamline 24-ID-C, using a Pilatus-6MF pixel array detector. The crystal was cryo-protected by a quick dip in a mixture of 65% mother liquor and 35% glycerol, and cryo-cooled to 100 K for data collection. A total of 720 diffraction images to 0.80 Å resolution were collected using 0.5° oscillations and a wavelength of 0.8266 Å. The data were processed using XDS/XSCALE^87^.

The crystal structure was determined using the direct methods program ShelxT^88^ and the model was refined using ShelxL^89^. After each refinement step, the model was visually inspected using the graphics program COOT^90^ and adjusted according to the 2mFo-DFc and mFo-DFc difference maps. Data collection and refinement statistics are reported in Supplementary Table 2. The X-ray crystallographic coordinates of the final model and the merged structure factors reported in this study have been deposited in the Cambridge Crystallographic Data Centre (CCDC), deposition number CCDC-1906101. These data can be obtained free of charge from the CCDC via www.ccdc.cam.ac.uk/data_request/cif.

### Powder X-ray diffraction (PXRD)

Fmoc-G-PNA conjugate at different concentrations were lyophilized, and crystals of the G-PNA tetramer were milled with pestle and mortar. Powder diffraction data were collected using a Bruker D8 Discovery X-ray powder diffractometer equipped with a Copper X-ray tube and a linear detector LYNXEYE-XE. The CuKb radiation was filtered by a Ni foil before the detector. Data collection was performed using a Bragg-Brentano geometry from 3 to 60°2q, with a step of 0.02°2q. The diffraction pattern was analyzed using the Pawley Method, as implemented in TOPAS software^91^.

### Fluorescence emission

Crystal emission spectra, lifetime, and quantum yield were measured using Fluorolog-3 (Horiba Scientific, Kyoto, Japan), at an excitation wavelength of λ_ex_ = 280 nm. Emission was recorded between 300–500 nm with excitation and emission slits of 5 nm. For lifetime measurements, 10,000 sequential decays were acquired, along with the instrumental response, in order to perform a fitting analysis. Data were analyzed using a DAS6 software with a multi-exponential model.

Fluorescence of solution samples was measured on a Horiba JobinYvon FL3-11 fluorimeter (Horiba Jobin Yvon, Edison, NJ, USA). Temperature control was achieved using a Peltier device (FL3). A quartz cuvette with a screw cap (to prevent evaporation of solvent), with an optical path length of 1 cm (Hellma Analytics, Müllheim, Germany), was used. The sample was stirred during the course of the experiment to maintain uniform temperature throughout the sample. The experiments were carried out by setting the excitation wavelength to λ_ex_ = 290 nm and recording the emission between 300–500 nm, with excitation and emission slits of 5 nm. Fluorescence experiment in the presence of Na and K cations (100 mM) were measured under the same condition.

ThT/TmPyP4 binding to Fmoc-G-PNA conjugate was evaluated, by preparing PNA samples of varying concentrations with 20 µM ThT (λ_ex_ = 440 and 290 nm) and 10 µM TmPyP4 (λ_ex_ = 433 nm) in 7/3 methanol/water (v/v).

### Fluorescent lifetime imaging microscopy (FLIM)

The fluorescence lifetime imaging was carried out using LSM 7 MP 2-photon microscope (Carl Zeiss, Weimar, Germany) attached to the Becker and Hickl (BH). The sample was excited at a wavelength of 1000 nm using Chameleon Ti:sapphire laser system with a repetition rate of 80 MHz. Fluorescent mages were collected through a Zeiss 20×1 NA water-immersion objective. Excitation and emission light were separated by a Zeiss dichroic mirror (LP 760) coupled to the instrument. Emission light was collected between wavelength of 300–500 nm through a hybrid GaAsP detector (HPM-100-40, BH, Berlin, Germany). The image acquisition time was set to 150–180 s to allow for sufficient number of photons to collect. Image analysis was done by SPCImage software (BH).

### ThT staining and confocal microscopy imaging

G-PNA crystals were mixed with 50 μL ThT (20 μM) and incubated at ambient temperature for 30 min. Ten microliter of the crystals were placed on a glass microscope slide. The stained samples were visualized using an LSM 510 META confocal laser scanning microscope (Carl Zeiss, Weimer, Germany) at excitation wavelength of 458 nm.

### Molecular docking

We performed molecular docking using AutoDock 4.3^92^ where a G-PNA tetramer was used as receptor and a ThT/TmPyP4 molecule as ligand. The G-PNA tetramer were rigid during conformational search, while for the ThT molecule, the bond between benzene ring and thiazole ring, and the bond between benzene ring and –N(CH_3_)_2_ group are rotatable.

### Young’s modulus measurement

Young’s modulus of the crystal was obtained using JPK, Nanowizard II (Berlin, Germany). Silica cantilever (PPP-SeIHR Nanosensor) with the spring constant of 10–130 N m^−1^, half-open angle of the pyramidal face of the tip was less than 10°, tip radius was about 8 nm. Sample was prepared by spreading G-PNA tetramer crystals over the surface of the quartz substrate (Supplementary Fig. 19). In a typical experiment cantilever was moved above the crystal with the help of optical microscope and it was brought to the crystal sample at a constant speed of 2 μm s^−1^ and held to the crystal surface at a constant force of 200 nN. After retracting from the crystal surface, the cantilever was moved to next spot to perform another cycle. The extend and retract force curves can be obtained at each spot of the dot matrix using the commercial software from JPK and analyzed with inbuilt software based on Igor pro 6.12 (Wavematrix 491 Inc.) and confirmed by manual fitting. 5–8 such dot matrix (5 µm × 5 µm, 600 pixels) were randomly set on each crystal. By fitting extend and retract force curves in the range of 10 nm from the contact point with custom-written Igor program, Young’s modulus of each spot can be calculated. The approaching curves were fitted using Hertz model,

1 $$F\left( h \right) = \frac{2}{\pi }\tan\, \alpha \frac{{{\mathrm{E}}_{{\mathrm{G}} - {\mathrm{PNA}}\,{\mathrm{tetramer}}}}}{{1 - v_{{\mathrm{G}} - {\mathrm{PNA}}\,{\mathrm{tetramer}}}^2}}h^2$$

where *F* corresponds to stress of the cantilever, *h* is the depth of the indentation, α is the half angle of the tip, *E*_G-PNA tetramer_ is the Young’s modulus of the crystals and ν_G-PNA tetramer_ is the Poisson ratio and it was set to 0.3. The obtained Young’s modulus was used to make the elasticity histogram.

### Point stiffness

In a typical point stiffness measurement, mechanical behavior of the crystal and cantilever can be considered as linear elastic. The measured point stiffness was obtained by fitting the extend and retract force curves. Since the crystal sample and the cantilever were considered as two springs, the measured point stiffness (*K*_meas_) comprises both stiffness of the cantilever (*K*_can_) and crystal (*K*_cry_). The point stiffness of the crystal was measured using the relation,

2 $$K_{{\mathrm{cry}}} = \frac{{K_{{\mathrm{can}}} \times K_{{\mathrm{meas}}}}}{{K_{{\mathrm{can}}} - K_{{\mathrm{meas}}}}}$$

### Computational methods

All calculations were performed using the generalized gradient approximation (GGA) exchange-correlation function of Perdew, Burke, and Ernzerhof (PBE)^93^, augmented by the Tkatchenko–Scheffler^94^ dispersive pairwise-terms, as implemented in the VASP projector-augmented planewave code^95^. A Brillouin zone sampling of 7 × 1 × 3 points was used along the reciprocal of the *a*, *b*, and *c* lattice vectors. A plane-wave cutoff of 800 eV was used to obtain converged stress calculations. The total energy was converged to 10^−7^ eV per unit-cell and all forces in the optimized structure were smaller than 10^−2^ eVÅ^−1^, with the stress optimized to be lower than the ratio between the minimal force and the lattice face area.

## Supplementary information

Supplementary Information

## Data Availability

The authors declare that the data supporting the findings of this study are available within the article and its Supplementary Information file, or are available from the authors upon request.
