# Supplementary material for: Mechanically rigid supramolecular assemblies formed from an Fmoc-guanine conjugated peptide nucleic acid
Source: Nat Commun. 2019 Nov 20;10:5256. doi: 10.1038/s41467-019-13250-x (PMC6868146; doi:10.1038/s41467-019-13250-x)
Supplement: Supplementary file 1 — Supplementary Information [file 41467_2019_13250_MOESM1_ESM.pdf]

SUPPLEMENTARY INFORMATION

Mechanically Rigid Supramolecular Assemblies Formed from an Fmoc-Guanine Conjugated Peptide Nucleic Acid

Vasantha Basavalingappa[1], Santu Bera[1], Bin Xue[2], Ido Azuri[3], Yiming Tang[4], Kai Tao[1], Linda J. W. Shimo[5], Michael R. Sawaya[6], Sofiya Kolusheva[7], David S. Eisenberg[6], Leeor Kronik[3], Yi Cao[2], Guanghong Wei[4], Ehud Gazit[1]*

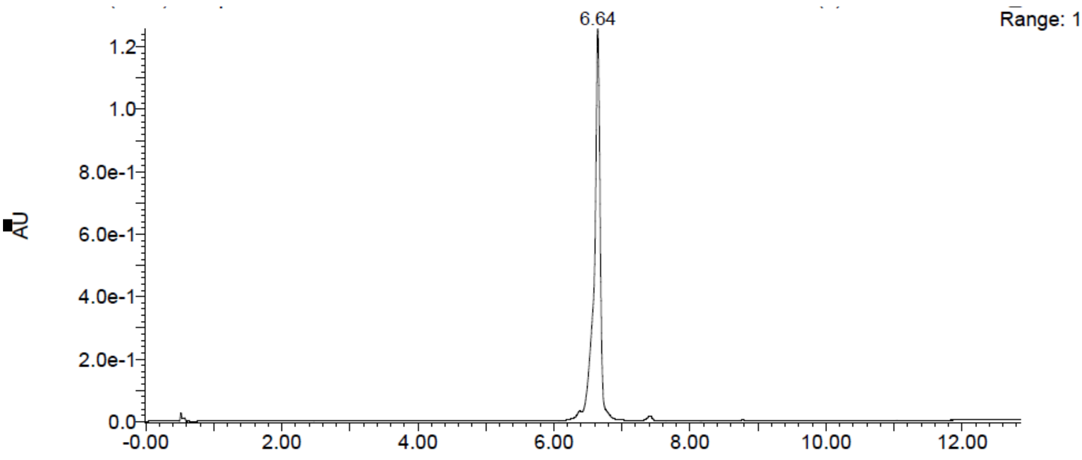

Supplementary Figure 1. LC profile of the pure adenine PNA-derivative.

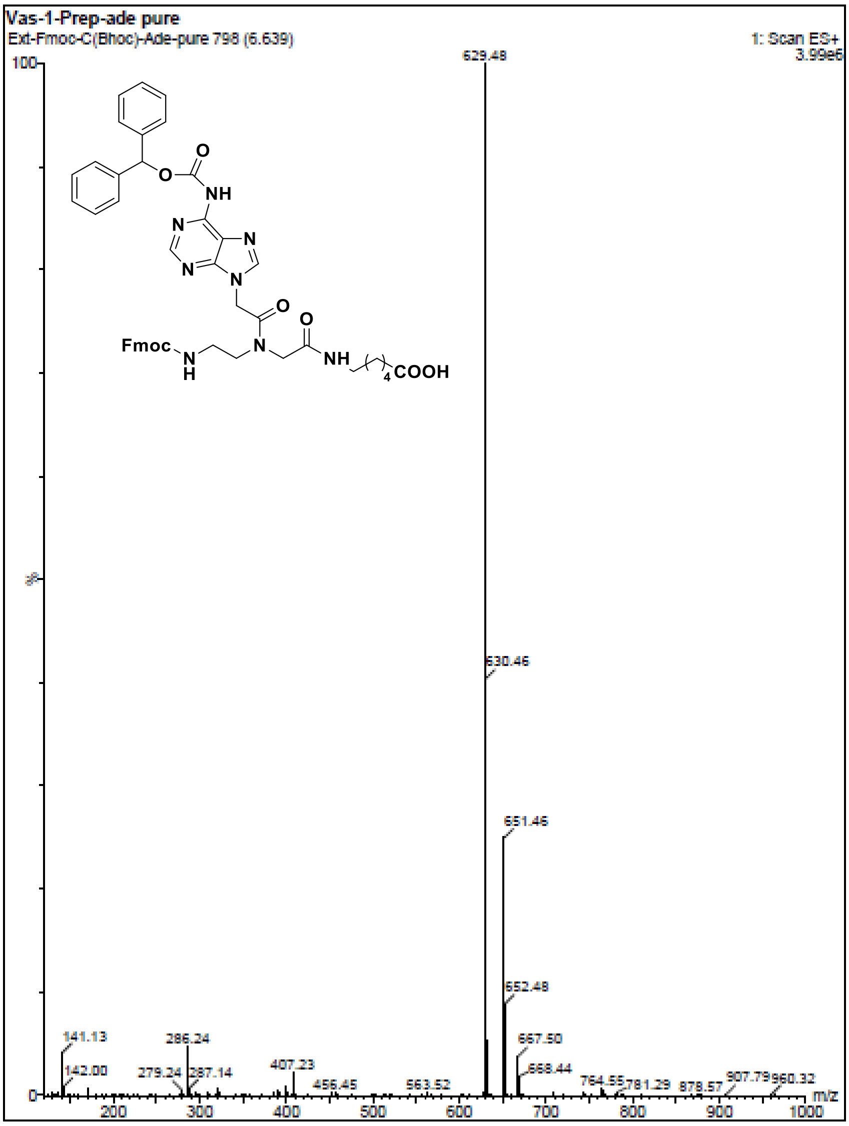

Supplementary Figure 2. Mass spectrum of the pure adenine PNA-derivative.

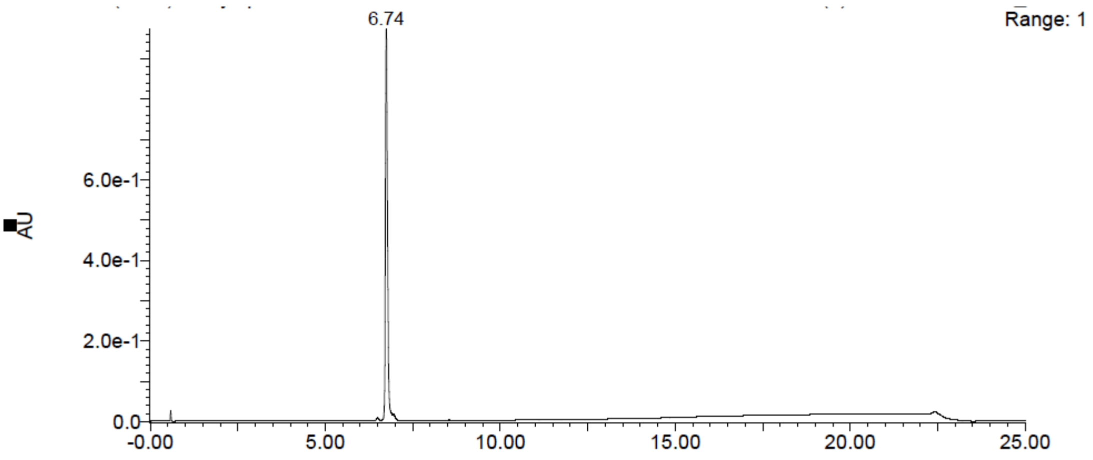

Supplementary Figure 3. LC profile of the pure cytosine PNA-derivative.

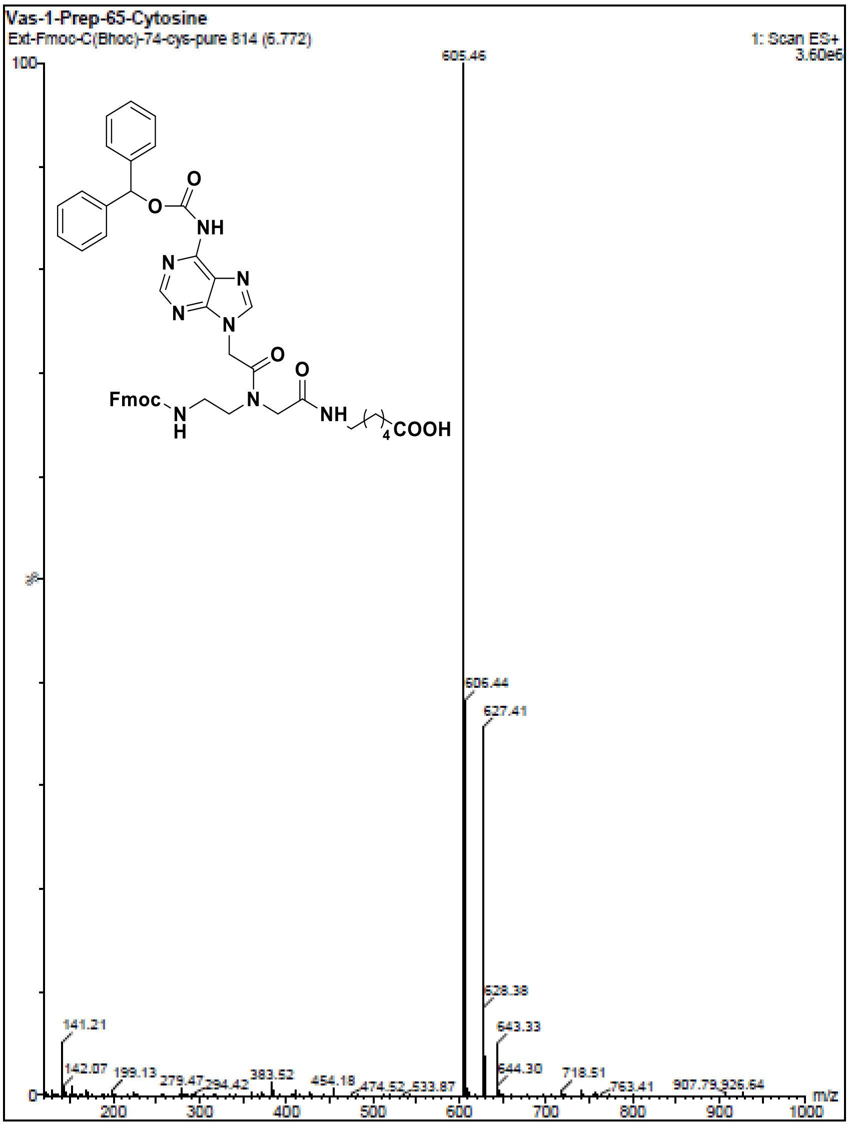

Supplementary Figure 4. Mass spectrum of the pure cytosine PNA-derivative.

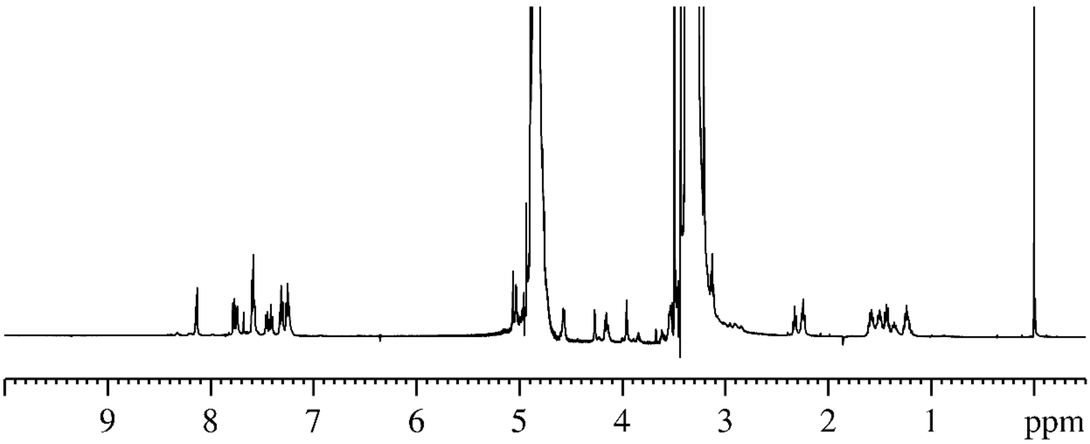

Supplementary Figure 5. A 500 MHz ^{1}H NMR spectrum of the adenine PNA-derivative in DMSO-d_6 and MeOH.

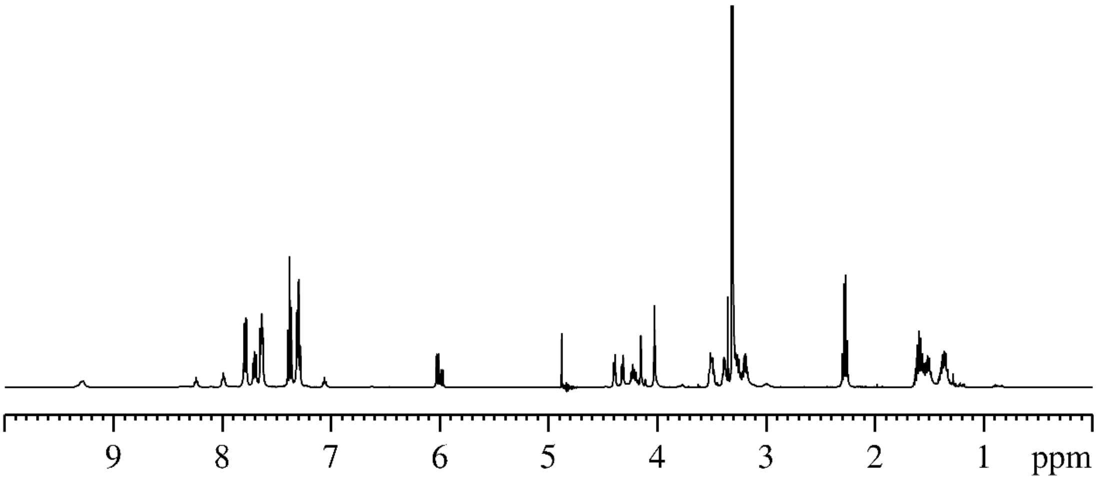

Supplementary Figure 6. A 500 MHz ^{1}H NMR spectrum of the cytosine PNA-derivative in MeOH-d3.

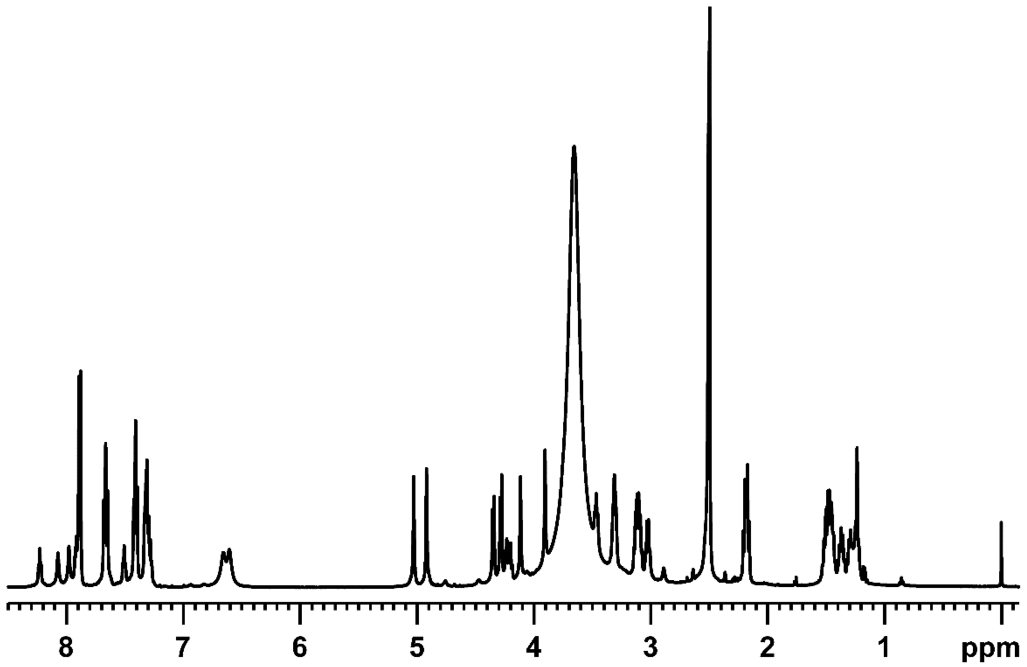

Supplementary Figure 7. A 500 MHz ^{1}H NMR spectrum of the PNA-quadruplex in DMSO-d_6.

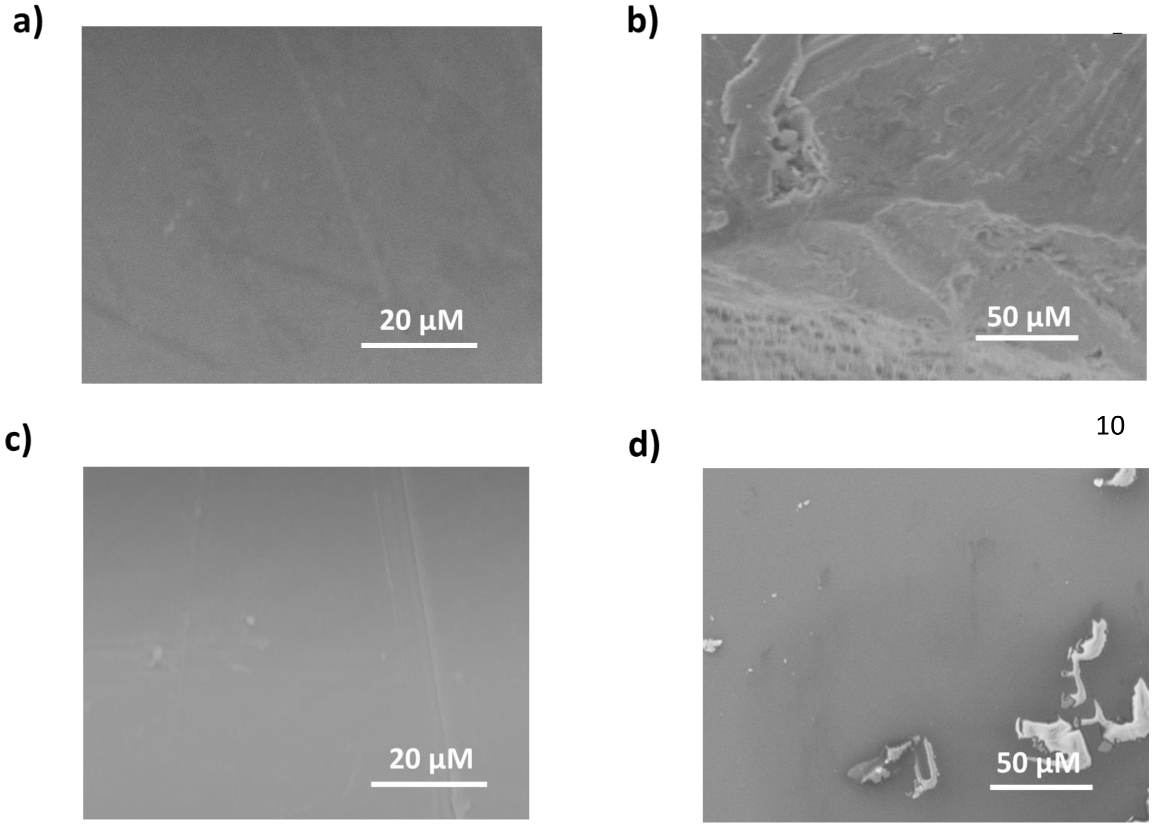

Supplementary Figure 8. Morphological characterization of adenine and cytosine PNA-derivatives. **(a, b)** SEM images of the adenine PNA-derivative at (**a**) 0.1 and (**b**) 0.5 mg/mL. (**c, d**) SEM images of the cytosine PNA-derivative at (**c**) 0.1 and (**d**) 0.5 mg/mL.

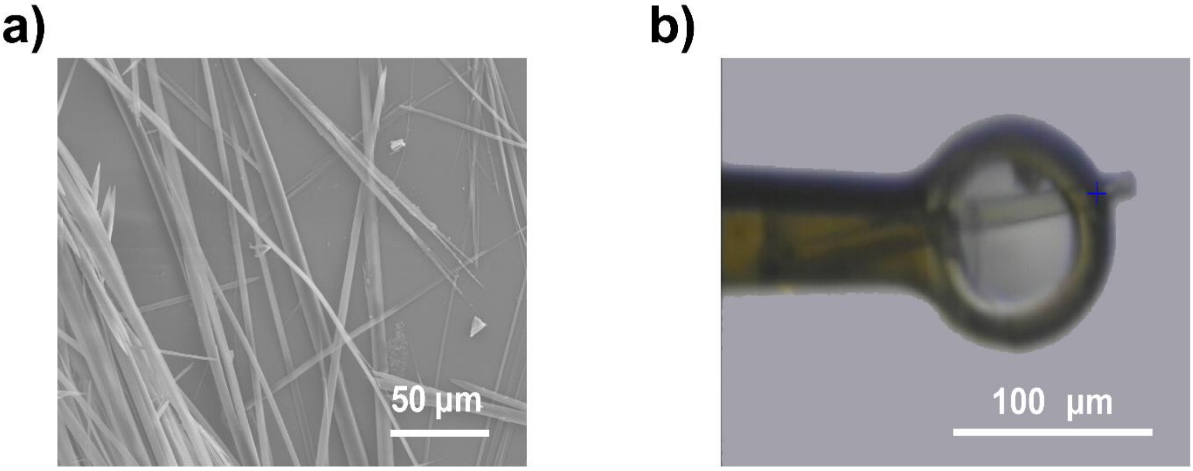

Supplementary Figure 9. Images of the PNA-quadruplex crystals. (**a**) SEM image. (**b**) View of the PNA-quadruplex crystal mounted and used for data collection.

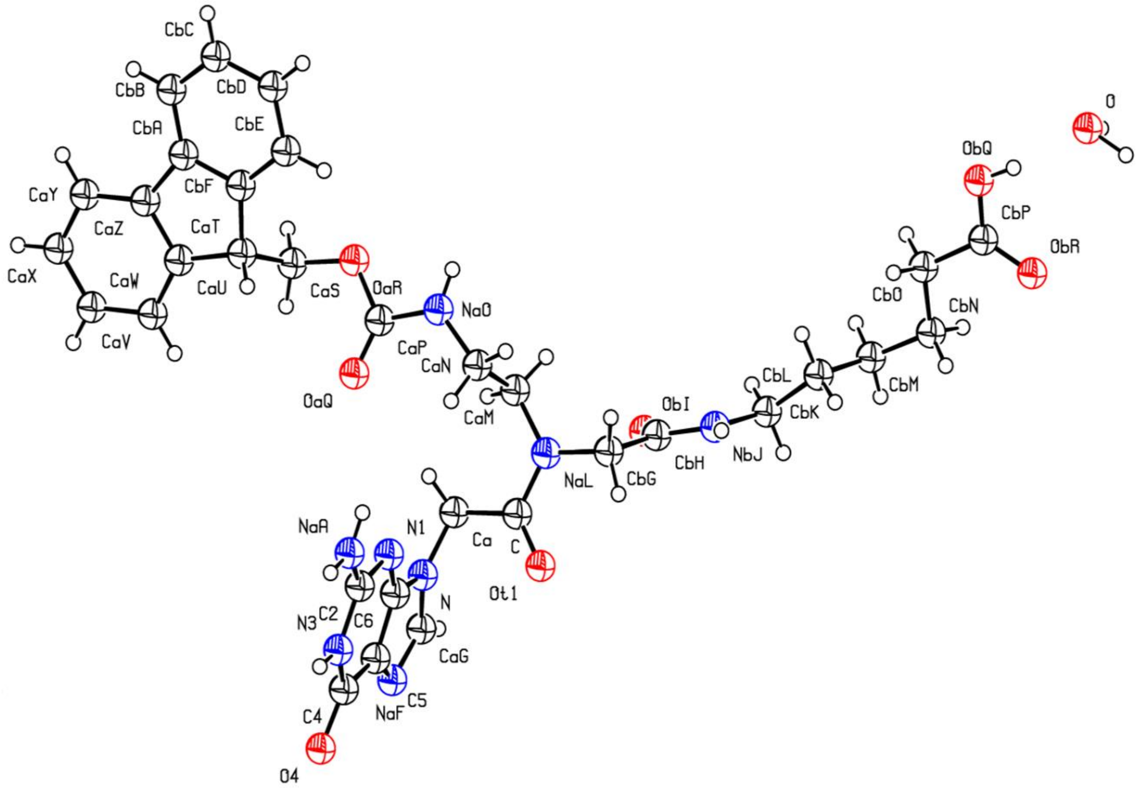

Supplementary Figure 10. ORTEP diagram of the PNA derivative in 50% probability ellipsoids, obtained from single crystal X-ray structure.

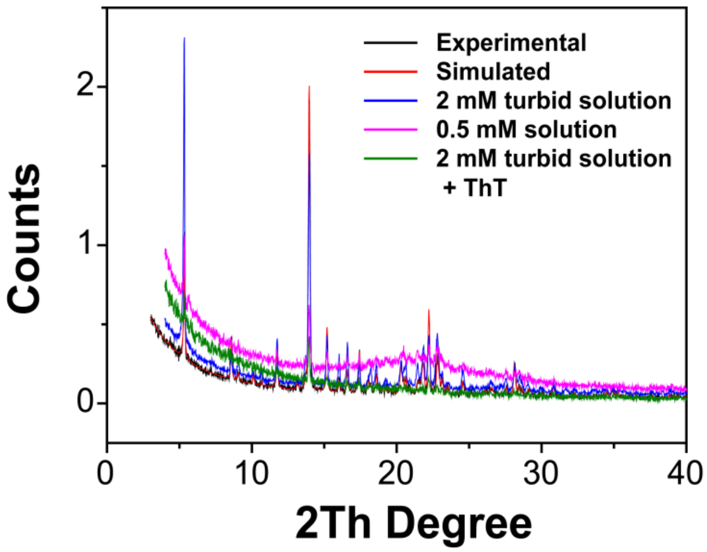

Supplementary Figure 11. Comparison of PXRD profiles of various self-assembled structural forms of PNA-quadruplex.

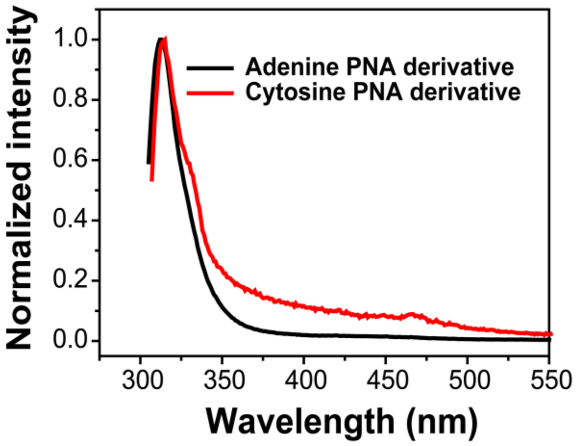

Supplementary Figure 12. Fluorescence emission spectra for adenine and cytosine PNA-derivative at 2 mM.

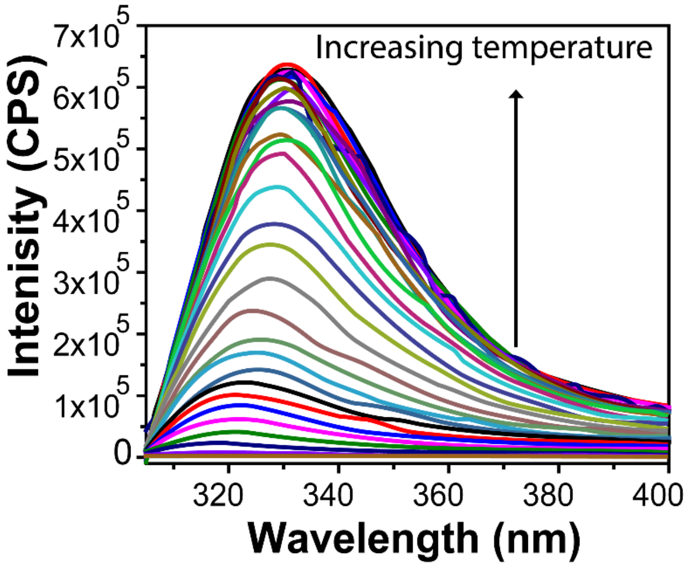

Supplementary Figure 13. Temperature dependence of the fluorescence emission peak at 330 nm, obtained from the PNA-quadruplex solution upon excitation at λ_{ex}=290 nm.

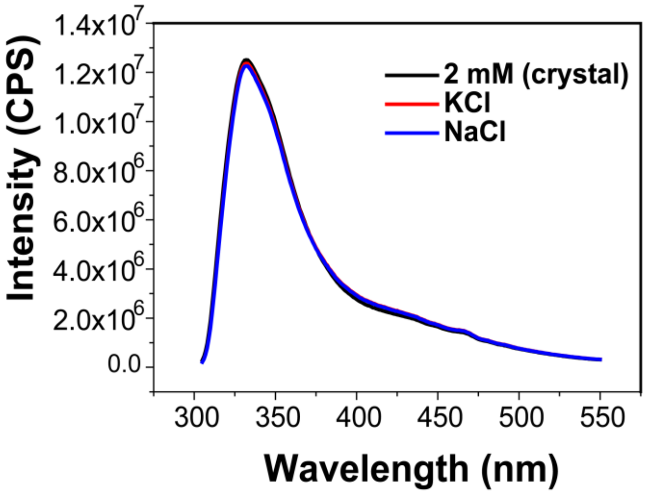

Supplementary Figure 14. Fluorescence emission spectra of PNA-quadruplex in the presence of 100 mM Na^+ or K^+ ions.

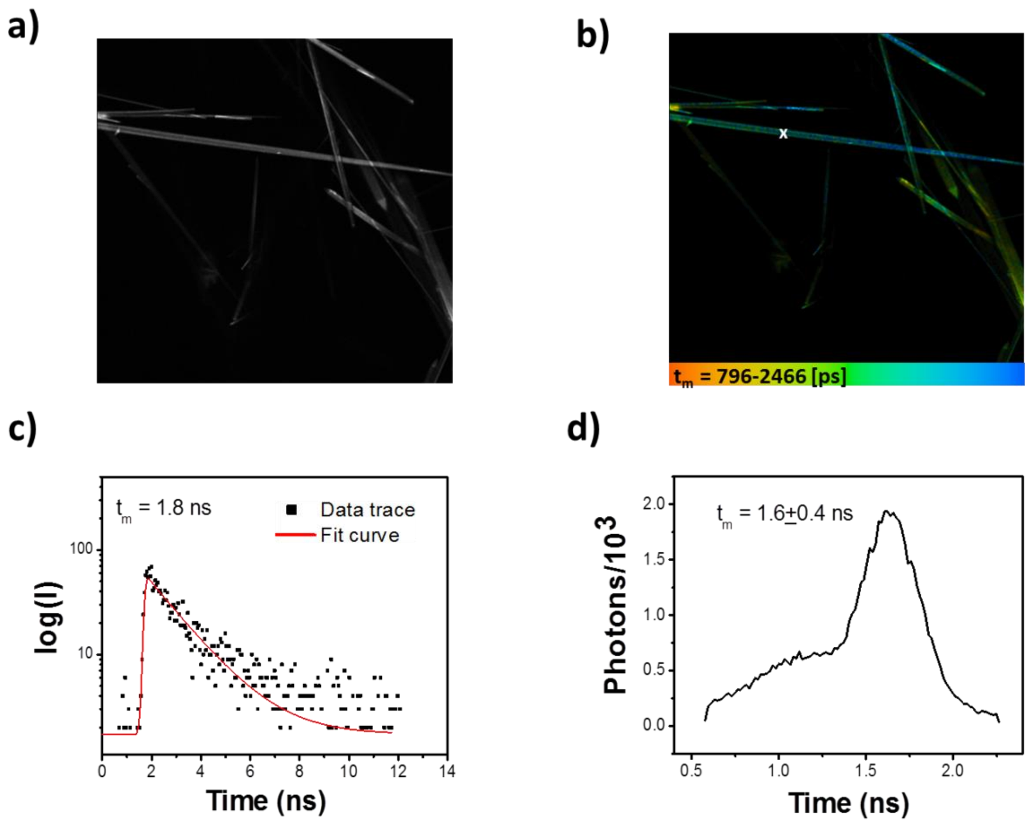

Supplementary Figure 15. FLIM characterization of PNA-quadruplex. (**a**) Bright field and (**b**) Fluorescence image of the PNA-quadruplex crystals. (**c**) Representative fluorescent emission decay curve at the white point in the FLIM image, showing a lifetime of ~2.0 ns. (**d**) Statistical fluorescence lifetime distribution of the crystals.

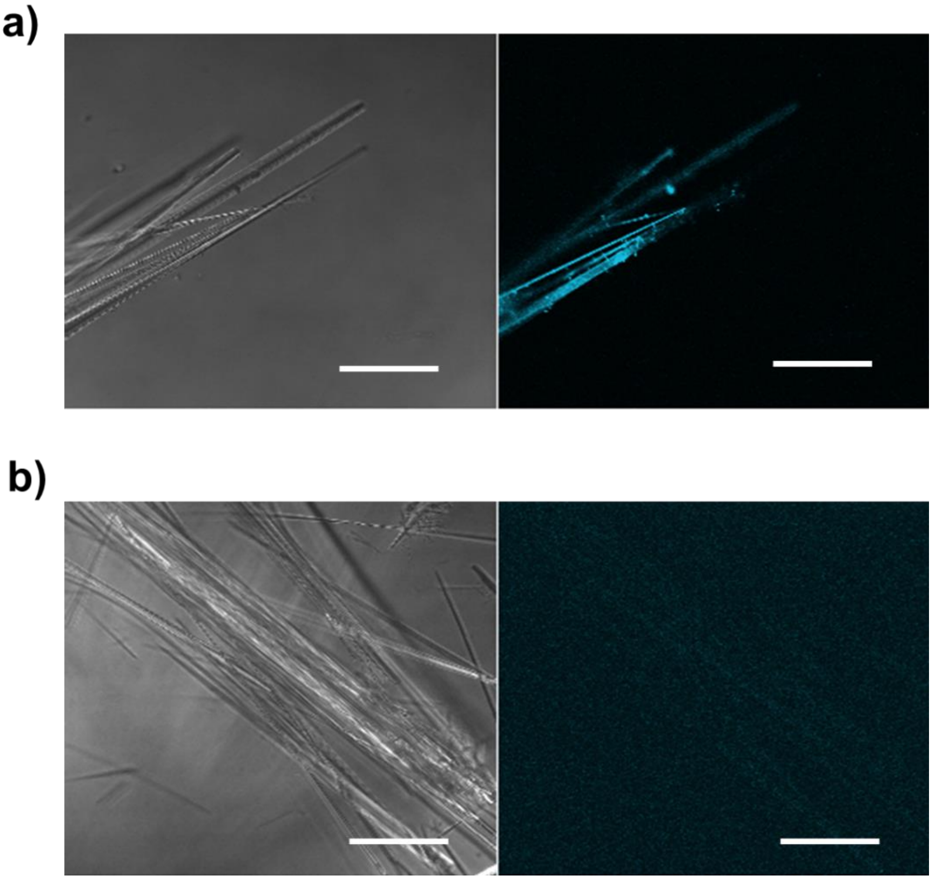

Supplementary Figure 16. Confocal fluorescence microscopy images of the PNA-quadruplex (**a**) with ThT, (**b**) without ThT. (left panel: bright field; right panel: fluorescent image, λex = 458 nm, scale bar 50 μM).

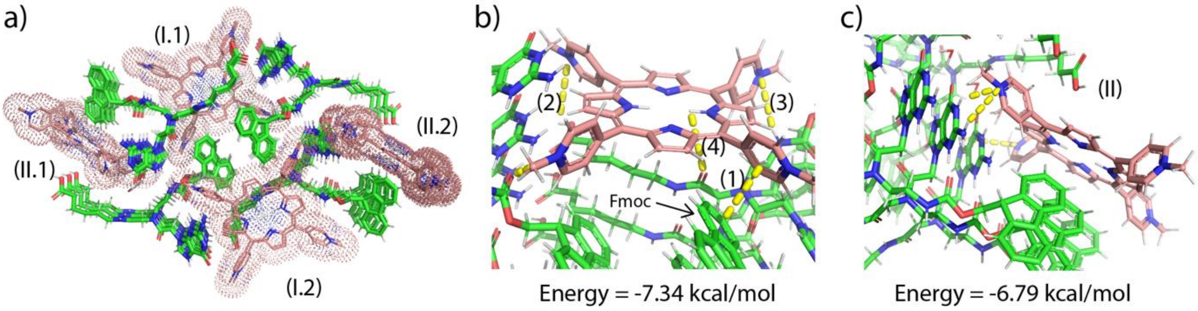

Supplementary Figure 17. Results of molecular docking of TMPyP4 to quadruplex inspired PNA-assemblies. (**a**) Binding sites of the first thirty energy-optimized docking pattern. The conformations are clustered into two groups: I.1 and I.2 are equivalent due to the crystal symmetry, so are II.1 and II.2. (**b-c**) Interactions responsible for molecule binding in the first (**b**) and the second (**c**) conformation clusters.

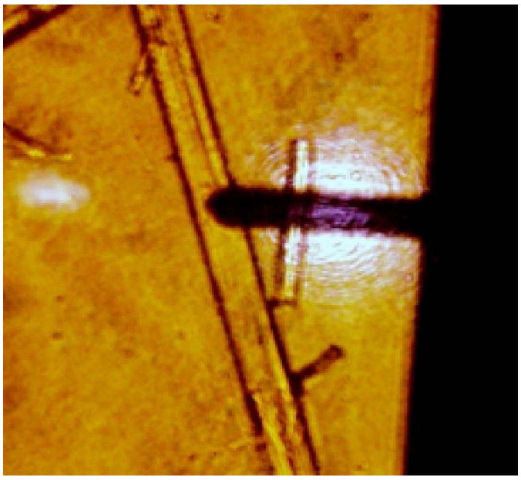

Supplementary Figure 18. Typical IT-AFM image. The crystals were fixed to the surface of the quartz substrate and the cantilever was moved to the crystal surface while monitoring under an optical microscope.

Supplementary Table 1. Crystallographic and computed lattice parameters for the PNA-quadruplex crystals, in Å. Values in parentheses indicate the relative error in the computed lattice parameters, with respect to experiment.

	$\vec{a}$	$\vec{b}$	$\vec{c}$
Experimental	4.74	32.86	20.48
TS-vdW DFT	4.66 (-1.7%)	32.63 (-0.7%)	20.33 (-0.7%)

Supplementary Details 1. Calculation of elastic constant tensor. 6 different distortions were applied to the unit cell, where, 1-6 are xx, yy, zz, yz, xz, and xy in the Voigt notation. These are:

$$\begin{pmatrix} 1+\delta_1 & 0 & 0 \\ 0 & 1 & 0 \\ 0 & 0 & 1 \end{pmatrix}, \begin{pmatrix} 1 & 0 & 0 \\ 0 & 1+\delta_2 & 0 \\ 0 & 0 & 1 \end{pmatrix}, \begin{pmatrix} 1 & 0 & 0 \\ 0 & 1 & 0 \\ 0 & 0 & 1+\delta_3 \end{pmatrix}, \begin{pmatrix} 1 & 0 & 0 \\ 0 & 1 & \delta_4 \\ 0 & 0 & 1 \end{pmatrix}, \begin{pmatrix} 1 & 0 & 0 \\ 0 & 1 & 0 \\ \delta_5 & 0 & 1 \end{pmatrix},$$

$$\begin{pmatrix} 1 & \delta_6 & 0 \\ 0 & 1 & 0 \\ 0 & 0 & 1 \end{pmatrix}$$

For each distortion, 5 strain (δ) values (0, ±0.005, ±0.01) were applied to calculate stress. The elastic tensor was obtained from numerical fitting of the obtained stress-strain curves.

Supplementary Table 2. PNA-quadruplex data collection and refinement statistics.

**Data collection**	**PNA-quadruplex**
Space group	$P2_1/c$
Cell dimensions	
a, b, c (Å)	4.74, 32.9, 20.5
A, β, γ (°)	90.0, 91.9, 90.0
Resolution (Å)	0.80 (0.82-0.80)
R_{sym}	0.048 (0.139)
$I/\sigma I$	17.2 (5.0)
$CC_{1/2}$	99.9 (98.8)
Completeness (%)	89.4 (72.9)
Redundancy	4.6 (3.1)
**Refinement**	
Resolution (Å)	0.80
No. reflections	5271
R_{work}/ R_{free}	0.080/0.085
No. atoms	
PNA-2 (including hydrogen)	83
Water	1
B-factors ($Å^2$)	
PNA-2	4.6
Water	5.7
R.m.s deviations	
Bond lengths (Å)	0.022
Bond angles (º)	2.2

*Highest resolution shell is shown in parenthesis.
